# Viral infection changes the expression of personality traits in an insect species reared for consumption

**DOI:** 10.1038/s41598-022-13735-8

**Published:** 2022-06-09

**Authors:** Matthew Low, Isak Eksell, Anna Jansson, Åsa Berggren

**Affiliations:** 1 Department of Ecology, Swedish University of Agricultural Sciences, Uppsala, Sweden; 2 Solskensvägen 12, 81541 Tierp, Sweden; 3 Department of Anatomy, Physiology and Biochemistry, Swedish University of Agricultural Sciences, Uppsala, Sweden

**Keywords:** Ecology, Zoology, Diseases

## Abstract

Disease-induced personality change results from endogenous and adaptive host responses or parasitic manipulation. Within animal husbandry systems understanding the connection between behaviour and disease is important for health monitoring and for designing systems considerate to animal welfare. However, understanding these relationships within insect mass-rearing systems is still in its infancy. We used a simple repeated behavioural-emergence test to examine parasite-induced differences in group personality traits in the house cricket *Acheta domesticus*, by comparing the behaviours of 37 individuals infected with the *Acheta domesticus densovirus* (AdDV) and 50 virus-free individuals*.* AdDV-infected animals had a much lower emergence probability, longer times until emergence, and did not change their behaviour with experience compared to the virus-free animals. AdDV-infected animals also had lower variation in their probability of emergence within the population, most likely related to animals displaying a relatively uniform sickness response. These infected animals also had higher variation in their response to experimental trial experience; this greater variation resulted from a difference between males and females. Infected females responded to experience in a similar way as virus-free animals, while AdDV-infected males showed a response to experience in the opposite direction: i.e., while all other groups reduced emergence time with experience, infected males always increased their mean emergence time as trials progressed. Our results are important not only in the context of animal personality research, but also with regards to creating husbandry systems and disease monitoring within the insects-as-food industry that are considerate to both production traits and animal welfare.

## Introduction

Personality research in animals is a field of study examining consistent individual differences in behaviour, with most research focussing on individual variation in boldness, exploration, activity, aggressiveness or sociability traits^1^. While not only documenting the existence of personalities and correlations between personality traits (‘behavioural syndromes’) in animals from a wide variety of taxa (e.g., mammals, birds, fish, reptiles and insects^2^), early personality research also sought to understand the causes of individual behaviour patterns and fitness correlates in relation to resource competition and predation risk^3,4^. More recently, however, personality research in animals has begun to focus on evidence linking behavioural traits to disease susceptibility, the expression of host or pathogen phenotypes via these traits, and ultimately individual and population measures of fitness^5–8^.

Although personality is primarily defined by behaviours that show consistency within individuals, exposure to new conditions can alter how individual personalities are expressed^9–11^. Changes in the expression of personality traits appear likely in the context of infection by a pathogen, since illness is known to reorganise an organism’s behavioural priorities to combat infection^12–14^ and/or to facilitate disease spread^15,16^. Studies have examined how host personality traits are related to individual parasite loads^16,17^ and mitigating infection risk^7^. In this context it is also interesting to consider the perspective of infection status changing the expression of personality traits within individuals and populations^15^. Evidence from studies on farm animals focussing on ‘temperament’ or ‘coping style’ as proxies for personality traits^18^, also suggest how a change in disease status likely impacts on individual personalities (i.e., reduced activity, exploration, aggression and changed social interactions). This perspective of disease-induced personality change is not only important for understanding how adaptive phenotypes are expressed between hosts and diseases at the individual and population level, but also of immense practical application when applied to animals used in livestock systems where disease impacts on behaviour can have major effects on the handling, welfare, health surveillance and production capacity of these animals^18^.

Personality research in aquaculture suggests that risk-avoiding fish may suffer from reduced welfare and fail to thrive because they cannot adequately compete for resources in an intensive husbandry system^19^. This raises the broader question of how disease, which often influences behaviours related to competitiveness^6,12^, impacts on animal production and welfare more generally within animal husbandry systems. Farm animal research on coping styles has been clearly linked to individual abilities in coping with environmental challenges^18^*.* While this has rarely been explicitly linked to disease status, it could be interpreted in terms of disease infection as the challenge requiring ‘coping’ from. This allows us to predict where problems may surface at the interface of disease and animal personality within different vertebrate husbandry systems, but only if there is a good understanding of baseline animal behaviour, its interaction with rearing conditions, and how those animals specifically respond to illness. However, such information is not yet available for many ‘new’ livestock systems. One of these is the insects-as-food industry, where large-scale intensive rearing is in its infancy but is rapidly expanding^20^. Here we know relatively little about how personality traits in most commercially-reared insects are expressed (but see Ref.^21^), how disease-behaviour syndromes manifest in these insects, especially in the context of how these factors interact with insect husbandry and animal welfare within intensive rearing systems. Thus, if these alternative animal production systems are to be viable in terms of production, welfare and long-term sustainability, it is important that we begin to understand the basic patterns of how individuals and populations express personality traits when healthy and when sick^20^.

**Figure 1:**
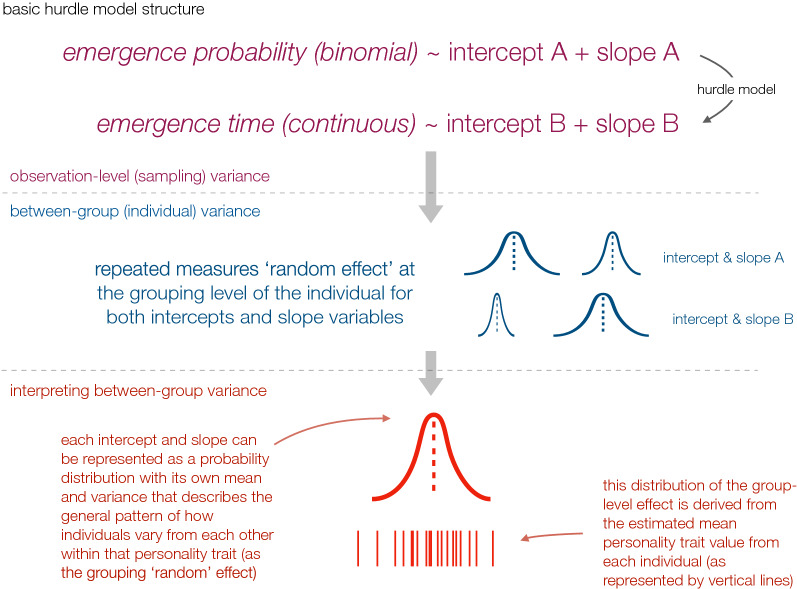
Schematic representation of the modelling approach used in this study to estimate the consistent individual-level differences in emergence behaviour. Observed emergence depended on a combination of a binomial and continuous process which we represented as a hurdle model: i.e. whether the individual emerged or not during the trial (binomial), and if it emerged, how long it took to leave the tube (continuous). Each of these processes was modelled as an intercept (average across all experimental trials) and a slope (change in probability or time with experience from each additional experimental trial). Because individuals were tested multiple times, these intercepts and slopes were modelled at a lower hierarchical level using the identity of each individual to estimate consistent differences between individuals. The Bayesian framework allowed us to examine these differences between individuals in two ways: (1) by extracting the posterior distributions that described the mean and range of the variation at the individual level, and (2) by directly estimating the mean for each individual’s ‘personality score’ for each of the model parameters (i.e. behavioural traits) of interest.

In this study we focus on the house cricket (*Acheta domesticus*), one of the most important species within the current insects-as-food industry^22^. Our interest was to examine patterns of individual behaviour using a classic emergence test (a behaviour correlated with boldness, exploration and activity personality traits; all of which are commonly related to the behavioural expression of health in animals^12,13^) and to contrast how aspects of this behaviour differed between groups of individuals known to be infected with *Acheta domesticus densovirus* (AdDV), and those that were certified to be virus-free. The AdDV is known to cause large-scale morbidity and mortality within populations, and has been flagged as a disease of major concern for insect rearers^23,24^. Thus, information about how sickness behaviour induced by AdDV is expressed by individuals and populations is a key aspect for assessing its threat to insect rearing and for finding husbandry measures to mitigate its impacts. Specifically, our aim was to examine how a personality trait (i.e., timing of emergence) varied between groups of individuals with and without AdDV during multiple experimental trials. Because insects are capable of learning^25,26^, and experience during an individual's development may influence its personality^4,27^, we also included experience in the trials as a key measure of the emergence behaviour: i.e., not only the average emergence behaviour, but also how this emergence behaviour changed with experience. Thus, our modelling approach described the observed emergence behaviour in terms of four main parameters, each with a clear biological interpretation (i.e. the binomial model intercept = the emergence probability; the gaussian model intercept = the time till emergence; the binomial slope = change in emergence probability per trial experience; the gaussian slope = change in time of emergence per trial experience); this allowed us to examine how each of these parameters changed between groups, independently of the other parameters (Fig. 1). There is also theoretical and empirical evidence to suggest that males and females may react in different ways to disease^28^. Thus, we also disaggregated the data for males and females to study these behavioural patterns of infection in relation to sex.

## Results

**Figure 2:**
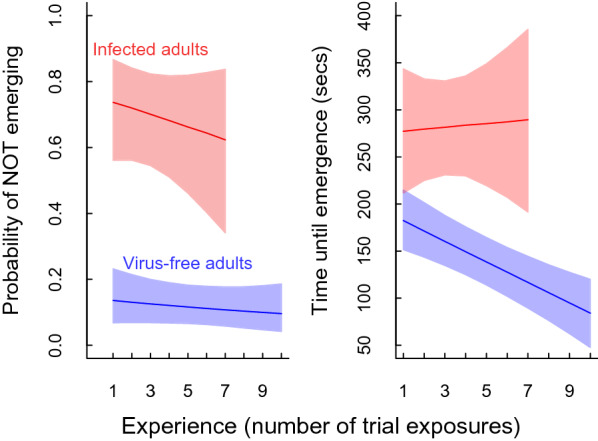
Predictions of the relationship between emergence probability (left panel) and time until emergence (right panel) for infected individuals (red) and virus-free individuals (blue) relative to the number of experimental trials an individual has experienced. Lines are means and shaded areas are 95% CIs as produced by our Bayesian hurdle model.

**Figure 3:**
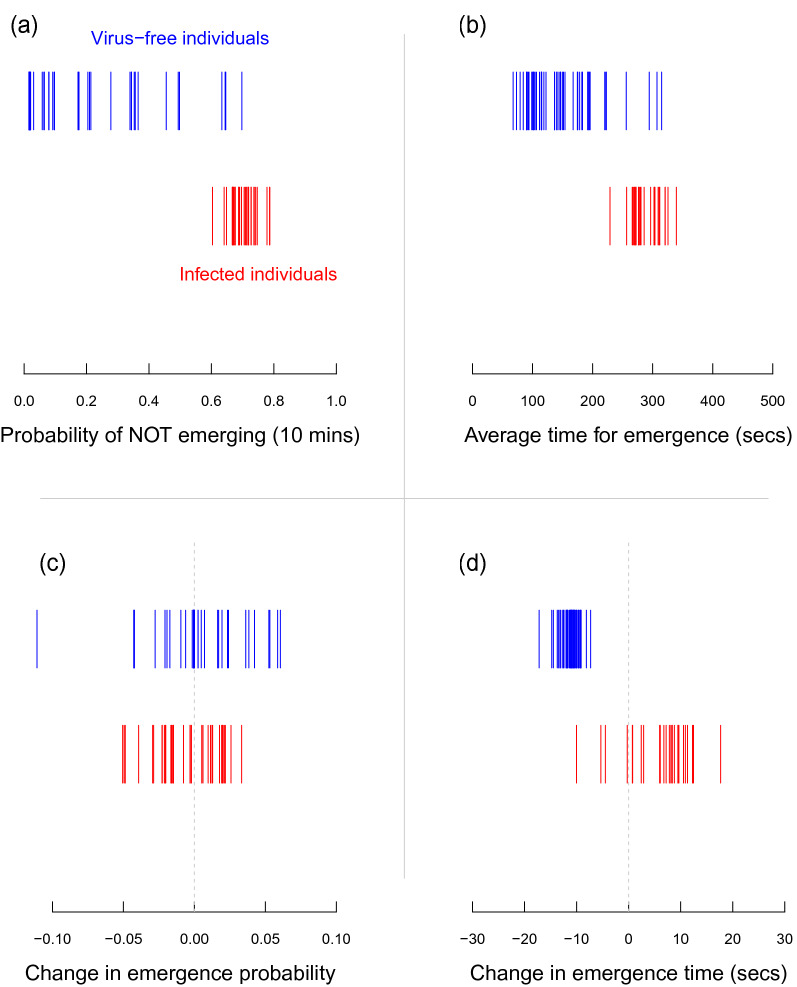
Estimated mean ‘personality scores’ for each individual cricket (vertical lines) for virus-free individuals (blue) and infected individuals (red), derived from the four model parameters that included an individual-level random effect: (**a**) binomial intercept, (**b**) continuous intercept, (**c**) binomial slope, and (**d**) continuous slope. See Table 1 for estimates of the raw parameters, Fig. 1 and Appendix S1 for a formal model description, and Fig. 4 for the individual personality scores to be disaggregated by sex.

There were clear differences between virus-free versus infected individuals in 3 of the 4 model parameters used to explain individual emergence behaviour (Table 1; Figs. 2, 3). The probability of not emerging from the tube during a 10-min trial was approximately four times higher for AdDV-infected individuals compared to virus-free animals (Table 1; Fig. 2). Latency time for those individuals that did emerge was also higher for infected animals, with latency decreasing with experience for virus-free individuals, but not for infected animals (Fig. 2).

**Table 1:** Means and standard deviations of the posterior distributions describing the individual-level ‘random effects’ within the model used to estimate individual personalities. Each of the four parameters in the hurdle model with a direct biological interpretation (i.e. binomial intercept and slope = probability of emergence and change in emergence probability with each experience; gaussian intercept and slope = time of emergence and change in emergence time with each experience) were represented at a lower hierarchical level to estimate each individual’s unique contribution to the observed variation. Thus these means and standard deviations describe the individual variation in personalities from the group of virus-free and infected individuals used in this study. We also show the posterior distributions for the difference between these groups for each estimated parameter (i.e. the mean difference in infected individuals from the virus-free group). From this derived parameter we could calculate the probability that an individual within the infected group had a higher ‘personality score’ for that parameter compared to an equivalent virus-free individual. High probabilities indicate that infected > virus-free, while low probabilities indicate that virus-free > infected (highlighted in bold). Probabilities in the mid range indicate that individuals these groups are likely to have a similar range of personality scores for that parameter.

	Virus-free	Infected	Difference (virus free—infected)	Probability (infected > virus free)
**Binomial intercept**^a^				
Mean	− 2.49 ± 0.46	0.845 ± 0.283	− 3.33 ± 0.54	**> 0.999**
st. dev	2.20 ± 0.476	0.661 ± 0.42	1.54 ± 0.64	**0.012**
**Binomial slope**^a^				
Mean	− 0.032 ± 0.09	− 0.046 ± 0.13	0.014 ± 0.163	0.455
st. dev	0.382 ± 0.133	0.328 ± 0.212	0.054 ± 0.25	0.389
**Gaussian intercept**				
Mean	149 ± 13.5	284 ± 29	− 135 ± 32	**> 0.999**
st. dev	75.1 ± 12.1	64.3 ± 34.7	10.8 ± 36.6	0.366
**Gaussian slope**				
Mean	− 10.8 ± 2.7	2.52 ± 13.4	− 13.3 ± 13.6	0.843
st. dev	5.33 ± 3.55	26.1 ± 15.4	− 20.7 ± 15.7	**0.916**

^a^At the logit scale.

By examining the underlying probability distributions explaining the personality differences between individuals (Fig. 1), similar patterns could be seen (Table 1; Fig. 3). However, here we could also quantify whether these personality traits differed not only in their mean, but also in their variance. Here we see the most probable differences between virus-free versus infected individuals is in the mean of the binomial intercept (> 0.99), gaussian intercept (> 0.99) and gaussian slope (0.84); and for the variance (calculated as standard deviation) we see the highest probabilities for between-group differences in the binomial intercept (0.99) and gaussian slope (0.92). Thus, not only did infection with AdDV likely change the mean expectation of individual personalities for three of the parameters, it also changed the variance or range of personalities in the population (decreased personality range for the probability of emergence, increased personality range for the effect of experience on change in emergence time; Table 1; Fig. 3).

**Figure 4:**
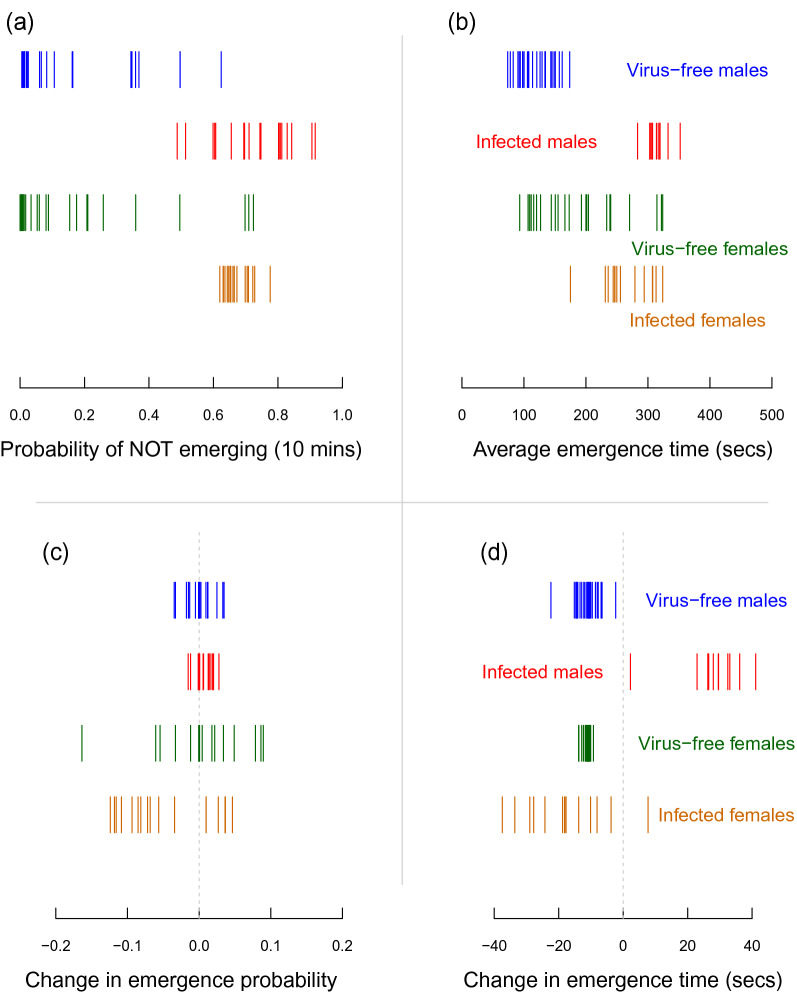
Estimated mean ‘personality scores’ for each individual cricket (vertical lines) for virus-free individuals and infected individuals, disaggregated by sex. From the top in each panel: blue = virus-free males, red = infected males, green = virus-free females, and brown = infected females. These estimates were derived from the four model parameters that included an individual-level random effect: (**a**) binomial intercept, (**b**) continuous intercept, (**c**) binomial slope, and (**d**) continuous slope.

By disaggregating the data based on sex, we could further examine whether any of these differences in personality traits between virus-free and AdDV-infected individuals differed in their effects for males compared to females (Fig. 4). Here the most striking difference was for the changes in emergence time with experience (Fig. 4d); all infected male individuals showed a general trend to increase their latency times, while all-but-one of the infected females reduced their emergence times with experience.

## Discussion

Parasitic infections, including viruses like AdDV, are often related to changes in insect host behaviour, and may range from exaggerations of current behaviours to the expression of completely novel traits^29^. While these behavioural changes are often not explicitly quantified in terms of ‘personality’, it is clear from a wealth of studies related to sickness behaviour that personality scores of activity, boldness or sociality will be influenced by general responses to infection such as depression and lethargy^12^. Here, whenever behavioural changes are observed subsequent to infection, it is worth considering if these changes serve some kind of function, and who benefits from their expression^30^. First there is the question of whether the changes in behaviour result from a host adaptation, where the host's resources are being conserved and redirected into fighting the infection; or is it because of the parasite manipulating the host into increasing the chance of spreading infection^12,30^? Second is when the behavioural response is a host adaptation, does this response result from mechanisms to avoid or fight the infection (so-called ‘resistance’ to infection), or does it result from an alteration in the individual’s life history to maximise its fitness without controlling the infection (so-called ‘tolerance’^31,32^). Answering these questions not only has major implications for understanding how organisms adaptively respond to disease, but has potentially important consequences for how we manage animals in captivity.

Parasitic infections likely cause multiple behavioural alterations in their hosts^29^. Here the simple emergence trait we measured could be considered as a combination of multiple components comprising the individual’s behaviour or personality within a group; each of which potentially could be influenced by a response to infection. Of the eight possible components we examined (i.e., the four means and four variances that described the random intercepts and slopes relating to biologically-interpretable individual personality traits), five of these showed high probabilities of differences between the virus-free animals and those infected with AdDV. Infected animals had a much higher probability of remaining within the tube during the experiment and slower emergence times compared to virus-free animals. In addition, AdDV-infected animals did not show any clear response to trial experience, while the virus-free animals were quicker to emerge over time (Fig. 2). These differences in the mean individual response likely result from the infected animals exhibiting sickness behaviour (i.e., lethargy and depression^12^) because of a reorganisation of their motivation^14^. However, since it is also known that crickets dying from AdDV infection often have an empty gut indicative of reduced feed intake^23^, and that temporary starvation increases shelter use in crickets^33^, it is possible that these behavioural changes are related to a starvation reaction rather than a direct disease response.

Interestingly we also observed infection-related differences in the variation of individual personality scores within the groups. In Fig. 3 it can be clearly seen that AdDV-infected individuals show very little variation from each other in their mean emergence probability or latency; whereas virus-free individuals have a much greater variation within the population. This difference is also likely related to a host response, whereby the sickness syndrome induced by infection limits the expression of personality traits attached to activity and boldness. These changes in behavioural expression could be a direct adaptive response by the insect to channel resources into its immune response, or an indirect adaptive response to energy conservation resulting from viral replication reducing the efficiency of energy intake. Of course, it cannot be ruled out that this reduction in behavioural variation results from some adaptive manipulation by the parasite on the host. While it might be expected under a viral manipulation scenario that host activity and sociality increases after infection to increase viral spread^29,34^, it is possible that the increased sheltering in a gregarious species^33^ could promote viral transmission. Finally, we also observed that AdDV-infected individuals had a much greater variance in their response to experience during the experimental trials. While the virus-free animals all showed a response to experience by reducing their latency during the trials, the AdDV-infected animals showed a wide variation in responses with some reducing their latency, while many increased it during the course of the experiment. This possibly reflects that these animals were getting sicker over time, and the more severe symptoms of the sickness response overshadowed any potential learning they might have gained by experience. However, by looking at the sex-disaggregated results (Fig. 4d) we can see that this larger variation in latency from the AdDV-infected individuals arises primarily from a response difference in sick males. Here the sick females continued to behave in a similar way as the virus-free animals (i.e. they reduced their latency with experience), while all of the AdDV-infected males showed an increasing latency with experience (or time). There are well-known sex effects on the expression of disease^28^; here we see a clear demonstration of this, with infected males seemingly less resistant to the progressive effects of infection, or its effects on learning, than females. The mechanism driving this difference between males and females most likely relates to sex-specific differences in ‘sickness syndrome’ expression^12–14^, with males simply becoming less active as the disease progresses (and hence taking longer to emerge), or them actively seeking shelter as a response to starvation^33^ induced by AdDV infection.

There are, however, two additional factors that we need to consider when interpreting the behaviours described above. The first is that the groups being compared are not from the same initial population. Ideally, we would have before-after control-impact data to clearly demonstrate the causal effect of infection on behaviour and personality. But logistical reasons necessitated that we compare animals from our own virus-free population being reared at the university, to animals of the same developmental stage and sex from a commercial rearer that was known to have endemic AdDV in their captive populations. This introduces the possibility that these groups differ in terms of their genetics and initial rearing conditions that are reflected in the behavioural differences we observe in this study. While we cannot discount these origin effects, it seems unlikely that this would be enough to explain what we observed in the trials. However, it does indicate that future studies should be based on infected animals derived from the same starting population as the virus-free animals. Second, is the concept of viral resistance versus tolerance by the host. We are assuming that the ‘sickness syndrome’ observed results from individuals trying to overcome the infection by channelling resources into their immune systems^12,35^. Because AdDV infection clearly has strong morbidity and mortality effects^23,24^, natural selection may instead of trying to resist the infection, instead attempt to maximise fitness outcomes via ‘tolerance’ of the virus^31,32^. This may be expressed via changes in feed intake quality and quantity or reproductive behaviour, as animals attempt to live with the parasite and maximise their fitness before dying. Because we did not compare fecundity, food preferences or sexual behaviours between the two groups, it is possible that the behavioural differences we observed are more related to viral tolerance than resistance as we are assuming. While this might seem an academic concern, it does have potentially significant impacts on animal husbandry and welfare.

Animals can resist or tolerate infection by seeking a new thermal optimum, often to raise their temperature^36^, but sometimes to cool it down^31^. By housing animals in environments without temperature and humidity gradients, we prevent this behavioural mechanism from operating (but this also prevents parasitic manipulation of behavioural fever^37^). Similarly, the ability to self-medicate or change the nutritional composition of the diet in response to infection (as a means to resist or tolerate chronic disease) is also limited in rearing facilities where often a single ‘balanced’ feed formula is provided. In this study our results show that the AdDV- infected crickets had different personalities to the virus-free animals, but we do not know how this manifests within a rearing situation where animals are given relatively homogenous ‘optimal’ conditions, compared to housing where they are able to choose their resting places (along thermal gradients for example) and feed type (where they may change the ratio of protein to carbohydrate when sick^31^). This not only has implications for animal production and population viability in captivity, but also touches on the issues of animal welfare. If insects can feel discomfort when their needs are not met, then we may need to start considering how sickness behaviour and the reorganisation of individual motivation when infected by a parasite impacts on their needs in a normal captive rearing situation. These concerns are not simple to quantify or address within mass-rearing systems, but such questions need to be asked not only from the perspective of animal welfare, but also for practical animal production.

Appropriate husbandry methods for the mass rearing of healthy insects are still being developed for an industry that is in its infancy^20^. These methods are being adapted from the experience of insects reared for pet feed and sterile insect production for pest control^38^, and there are serious challenges to create conditions that not only are suitable for healthy insect rearing, but that also offer opportunities for individuals to appropriately respond to stressors like pathogens. Based on our observations in this study, it is clear that benefits for the industry and animal welfare can be derived from behavioural experiments that examine individual reactions to changing infection status. For example, can choice experiments with regards to changing temperature and food preferences in sick animals inform the industry in ways to modify environmental factors to limit the spread and impact of disease in mass-rearing conditions? The industry today is focused on species that are still in the early stages of domestication selection, and so husbandry responses need to be considerate of adaptive behavioural responses in wild species. It is likely that conflicts will arise when attempting to create rearing situations that cater fully for the environmental variation needed for adequate expression of behaviours linked to health and welfare in insect species. But we need to first understand the needs of the individual insects, before we can make informed decisions regarding how to trade-off adaptive behavioural responses against constraints imposed by facility design geared towards production goals.

## Methods

### The animals

The house cricket *Acheta domesticus* (Orthoptera: Gryllidae), is a medium sized (body length 14–20 mm) omnivorous insect with a global distribution^39,40^. In temperate regions it is found in a range of habitats (e.g., compost heaps, hedgerows, meadows) and often close to urban settings^39,41^. Males stridulate to attract females, and nymphs reach adulthood after 7–14 stages^42^. Females lay their eggs in moist soil or similar material, and nymphs hatch after 2–3 weeks at 20 °C^39,41^. For the personality trials we used 50 virus-free adult crickets (26 males, 24 females) and 37 AdDV-infected adults (19 males, 18 females). The virus-free animals were captive reared at Swedish University of Agricultural Sciences and descended from wild-caught animals after approximately six generations in captivity. The virus-free animals were randomly selected from five different breeding lines to reduce the risk of personality traits being constrained by genetic origin. AdDV-infected individuals were sourced from a commercial Swedish rearer and were of comparable life-stage (i.e. adults) to the virus-free animals. All individuals were housed under controlled conditions with a 12 h lighting regime, temperature 30° C and relative humidity 40%, according to optimal recommended conditions for house crickets^43,44^. The commercial animals infected with AdDV were confirmed with the virus using in-house testing based on frass samples^45^; viral titres for all samples > 9 log IU/mL. The virus-free crickets were also tested and confirmed free of AdDV before the experiment using the same methods^45^.

### The virus

The Acheta domesticus densovirus (AdDV) is a member of the Parvoviridae virus family^46,47^ and infects *A. domesticus*^23,24,48^. The virus can also infect other cricket species, but has only been shown to be fatal to *A. domesticus*^49^. AdDV infection in cricket populations often results in widespread mortality and even extinction of local cricket populations^23,50^. Infected crickets show a range of symptoms, such as malnutrition, inhibited growth, reduced fecundity, paralysis and death^23,51^. Although the exact mechanism is unknown it has been shown that AdDV has a coding sequence for a phospholipase thought to be critical for cellular uptake of the virus^51^ and it might therefore, as suggested for other densoviruses, cross the intestinal epithelium to reach and replicate in underlying target tissues, disturbing midgut function^52^. The frequency and distribution of this virus in commercial insect rearing facilities are currently unknown, but could be widespread^53,54^. Frass from the AdDV-infected individuals used in the trials was tested and individuals were confirmed to be AdDV-positive (> 9 log IU/mL) using the established methods developed by Semberg et al.^45^.

### The experiment

We used a well-established behavioural measure (emergence from a tube) and consistent methodology during the experimental trials (based on Refs.^55–58^). The handling and housing of all individuals was the same between trials, whereby the crickets were kept in clear plastic containers with a netted roof to ensure air circulation at a constant 30° C and humidity of 40%. In each cage individuals had access to shelter and were provided with ad libitum food and water. Between experimental trials crickets were housed individually or in small groups, with all crickets able to see and hear the crickets in other housing containers. Each cricket in a group was individually marked on its thorax using a non-toxic insect paint (Kölner Vergolderprodukte, Germany). The housing and experimental trials were conducted in the same room to minimise handling; however, because of the risk of virus spread between the virus-free and infected individuals, their housing and trials were in separate facilities.

An experimental trial consisted of placing an individual cricket inside a black opaque tube (7 cm long with a diameter of 2 cm) with a lid on one end. Individuals were captured by ‘herding' the cricket into the tube by lightly tapping behind the animal in their housing cage. For transfer to the experimental cage a lid was placed over the open end of the tube. The tube was then placed horizontally in the trial arena: i.e., a cage measuring 18 × 18 × 10 cm that was based on previous experiments on boldness in related species^59^. Once placed in the experimental arena, the cricket in the tube was left for 2-min before the lid opposite to that where the cricket entered was removed. A plexiglass cover was placed on the roof of the arena to allow observation, but to minimise any auditory disturbances. The behavioural measure recorded during the trial was the time taken (in seconds) for the cricket to fully exit the tube. Observations were limited to a maximum of 10 min for each individual trial. This limit was determined based on previous experiments showing that if a cricket stays in the tube for longer than 10 min, it is likely to stay for an extended period of time^57^. The arena and tubes were cleaned with ethanol between trials to limit behavioural responses to smells from individuals in previous trials. Each individual was tested every 1–2 days for 7–10 experimental trials, with the same person responsible for all observations. Because of the higher mortality associated with the virus-infected individuals, to ensure sample sizes were reasonable, if one of these animals died between trials it was replaced by a new individual of the same sex.

### Statistical analyses

The individuals’ emergence behaviour created data structured by two processes: (1) if the individual emerged or not during the trial (a 1/0 variable), and (2) for emerged individuals, the latency for this emergence (a continuous variable between 1 and 599 s). Thus, to model the emergence data we used a binomial-gaussian hurdle model. This is a model structure that uses all data for modelling the binomial process (since all individuals either stay or leave the tube), but only uses the subset of individuals who left the tube during their experimental trial for modelling the gaussian process (Fig. 1). We initially considered a gamma distribution for modelling the continuous process, but the data were structured in a way that allowed a gaussian distribution to adequately represent the data (based on posterior predictive checks on model fit; see Appendix S1). For both the binomial and gaussian processes within the hurdle model we used the same linear model structure: i.e., an intercept to represent the mean emergence probability (binomial) or latency till emergence (gaussian), and a slope parameter to model the potential effect of experience on emergence behaviour (i.e., change in probability or latency per experimental trial). To account for the repeated sampling of individuals, we included ‘individual ID’ as a grouping variable (a.k.a. random effect) on the intercepts and slope terms being estimated within each part of the hurdle model (see Fig. 1 and Appendix S1 for full model structure). The model was implemented in a Bayesian framework in R^60^ using JAGS^61^ with minimally informative priors. All models were checked for convergence by visual inspection of stability and mixing of the chains, and fit using posterior predictive checks (see Appendix S1 for more details of model structures and other metrics).

For analyses of personality traits, consistent individual differences in behaviour are typically demonstrated by comparing the within- versus between-individual variances in the personality measures of interest (e.g. Refs.^62,63^). To do this, we took advantage of the hierarchical nature of our Bayesian model to directly estimate each individual’s specific personality score relative to all other individuals being tested. This was possible because the model's structure includes random intercepts as well as random slopes for each individual^32,63^. Thus, the repeated measures from each individual allowed us to not only statistically account for non-independent sampling of individuals, but subsequently could be used to provide an estimate for each individual’s ‘personality score’. These could then be summarised across the entire group of individuals being tested to generate the underlying mean and variance for the distribution of the individual personality scores (see Fig. 1). This also meant we could assess whether the general patterns of individual ‘personalities’ differed from group to group (e.g., virus-free versus AdDV-infected, or males versus females), either in terms of their mean value, or their variance. Because our model described emergence behaviour in terms of four main parameters, each with a clear biological interpretation (i.e. the binomial model intercept = the emergence probability; the gaussian model intercept = the time till emergence; the binomial slope = change in emergence probability per trial experience; the gaussian slope = change in time of emergence per trial experience), we could also examine how each of these parameters changed between groups, independently of the other parameters. These Bayesian posterior distributions are particularly useful for comparing estimates between groups because the difference between two groups (e.g., is the mean value of virus-free emergence time > infected?) can be calculated by simply subtracting one posterior distribution from the other. Subsequent interpretation is then based on the fact that all variables derived in this way are themselves probability distributions. Thus, parameters and derived variables where the posterior distribution do not overlap zero to a large degree indicate clear differences between groups^64^. Such flexibility allows for quantifying the direction and magnitude of any expected average change in personality as animals were compared as being virus-free or infected (or between males and females).

## Supplementary Information

Supplementary Information.

## Data Availability

The data are openly available from the Swedish National Data Service www.snd.gu.se under the data description ‘House cricket personality tests based on emergence behaviour in experimental trials’ https://doi.org/10.5878/s283-2r88.
