# Supplementary material for: Viral infection changes the expression of personality traits in an insect species reared for consumption
Source: Sci Rep. 2022 Jun 9;12:9503. doi: 10.1038/s41598-022-13735-8 (PMC9184467; doi:10.1038/s41598-022-13735-8)

Supplementary Appendix S1 for the Scientific Reports paper:

**Viral infection changes the expression of personality traits in an insect species reared for consumption**

by Matthew Low, Isak Eksell, Anna Jansson & Åsa Berggren

Contained in this appendix is:

1. Full Bayesian model formulation described, including priors

2. Outputs of posterior predictive model checks showing the fit of the model to the data

1. Bayesian hurdle model formulation for extracting estimates of the four main factors of interest: i.e. the binomial model intercept = the emergence probability; the gaussian model intercept = the time till emergence; the binomial slope = change in emergence probability per trial experience; the gaussian slope = change in time of emergence per trial experience (see also Fig. 1 for visual description of the model)

Model

emergence *_i_* ~ Binomial (prob *_i_* )

logit ( prob *_i_* ) = intercept.A *_j_* + slope.A *_j_*

intercept.A *_j_* ~ Normal (mu.int.A, sigma.int.A)

slope.A *_j_* ~ Normal (mu.slope.A, sigma.slope.A)

emergence time *_k_* ~ Normal (mu *_k_*, sigma )

mu *_k_* = intercept.B *_j_* + slope.B *_j_*

intercept.B *_j_* ~ Normal (mu.int.B, sigma.int.B)

slope.B *_j_* ~ Normal (mu.slope.B, sigma.slope.B)

#priors

mu.int.A ~ normal(0, 1000) #mean emergence probability

sigma.int.A ~ uniform(0, 10) #variance in emergence probability

mu.int.B ~ normal(200, 1000) #mean emergence time

sigma.int.B ~ uniform(0, 10) #variance in individual emergence times

mu.slope.A ~ normal(0, 1000) #mean experience effect emergence prob.

sigma.slope.A ~ uniform(0, 50) #variance experience emergence prob.

mu.slope.B ~ normal(0, 1000) #mean experience effect emergence time

sigma.slope.B ~ uniform(0, 50) #variance experience emergence time

2. Formal model fit using posterior predictive checks and comparing fit using Bayesian P. Here we simulate a dataset from the model parameters and likelihood distribution to check how well our model fits the data in terms of the predicted mean and coefficient of variation (CV). Here we want the simulated data (y-axis) to approximately centre on the observed data. The Bayesian P simply describes the proportion of simulated datasets that are above the observed data (and hence, 1-P is the number of datasets below). Ideally we want P to be close to 0.5 to show a good model fit (but anything between 0.1 - 0.9 is considered acceptable). Here we show the comparison of observed to model simulated data for both the binomial data, and the continuous data.
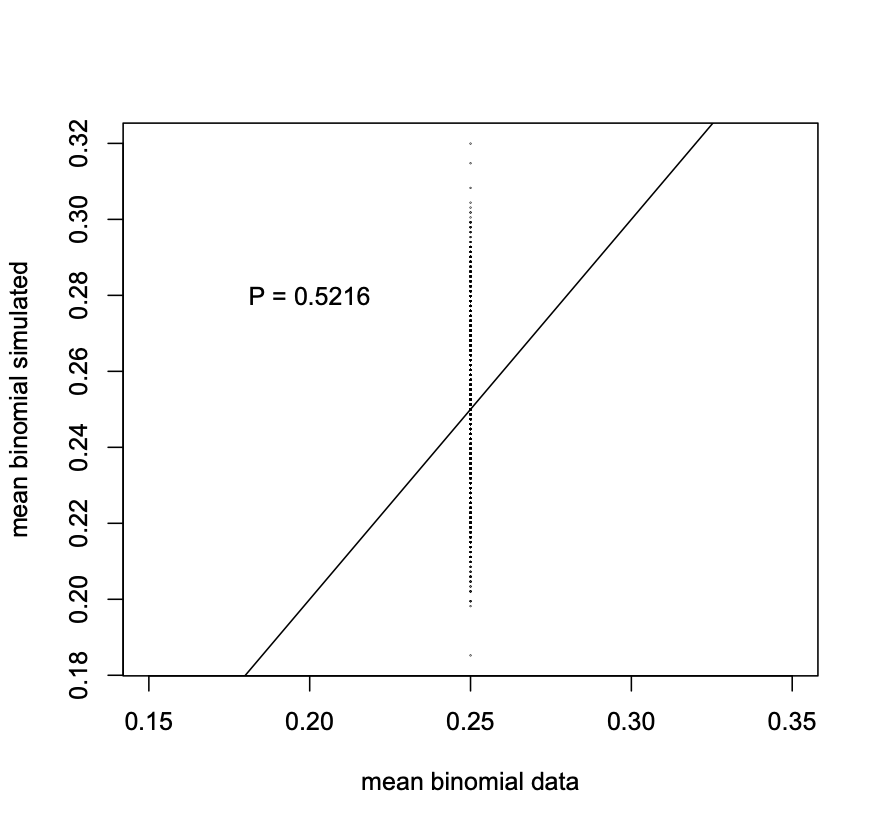

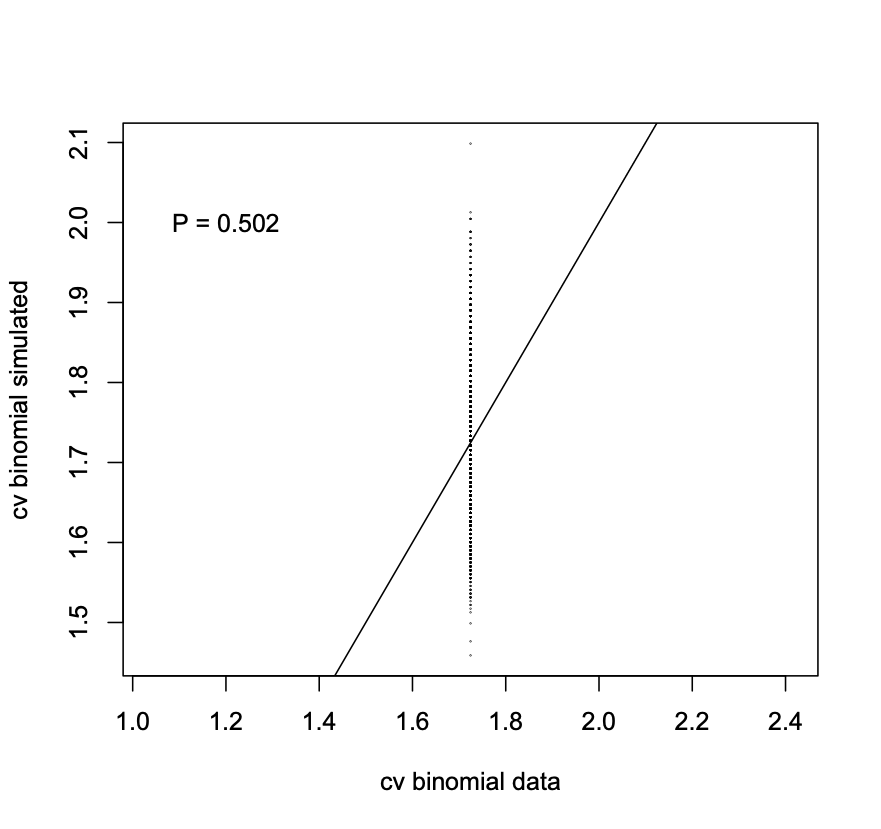

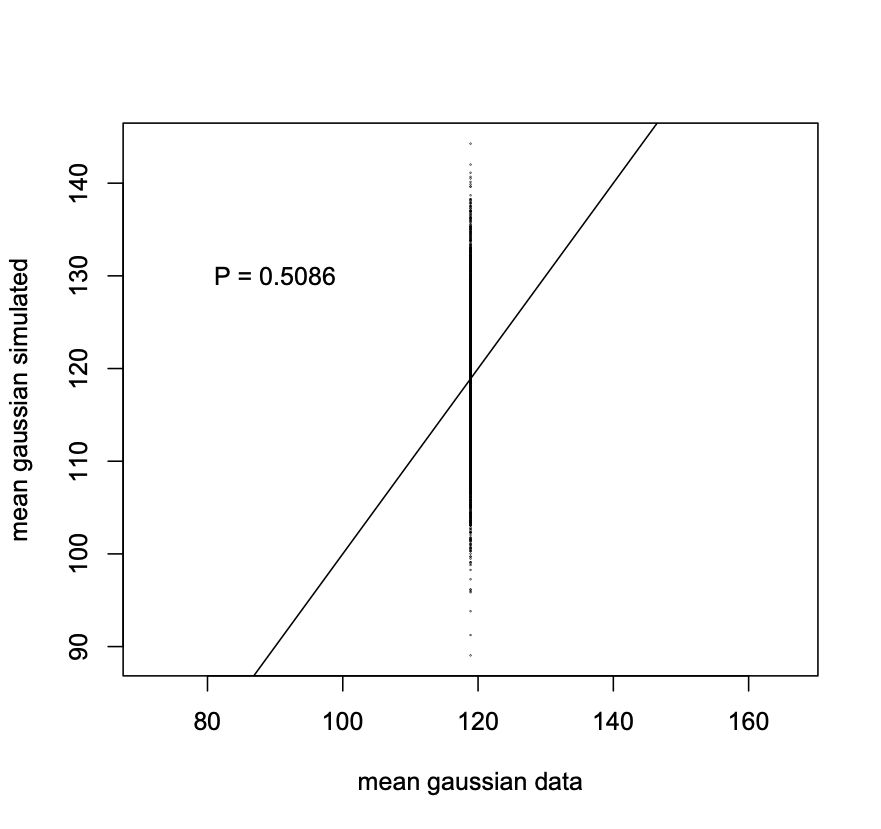

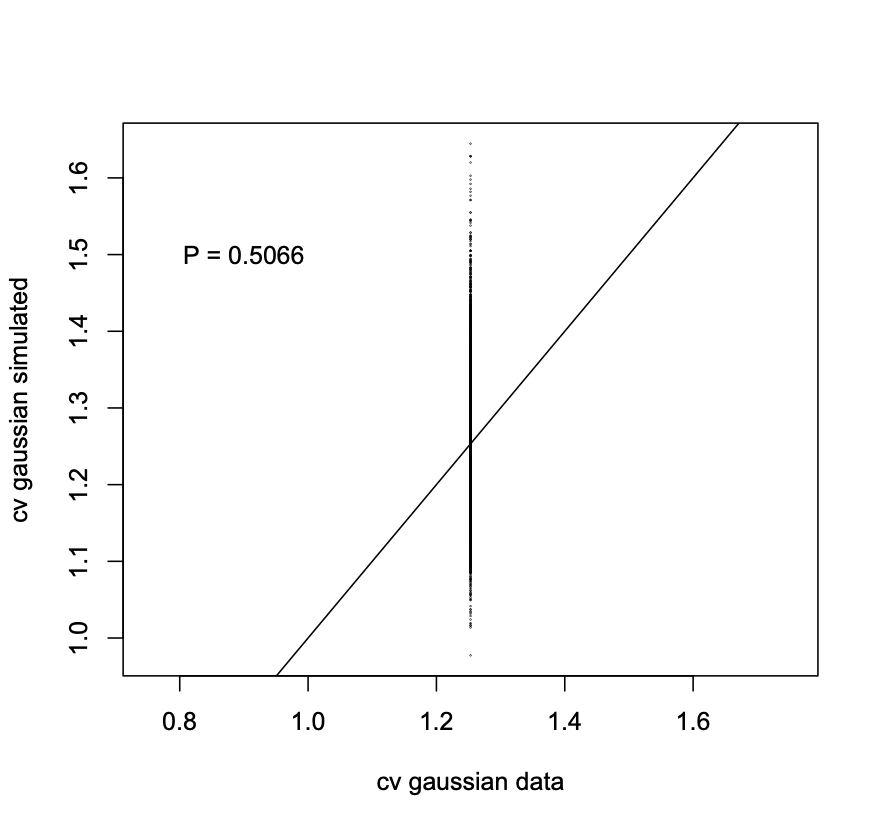

Supplement: Supplementary file 1 — Supplementary Information. [file 41598_2022_13735_MOESM1_ESM.docx]
